# A review on lung boundary detection in chest X-rays

**DOI:** 10.1007/s11548-019-01917-1

**Published:** 2019-02-07

**Authors:** Sema Candemir, Sameer Antani

**Affiliations:** Lister Hill National Center for Biomedical Communications, Communications Engineering Branch, National Library of Medicine, National Institutes of Health, Bethesda, USA

**Keywords:** Chest X-ray, Lung region detection, Region of interest detection

## Abstract

**Purpose:**

Chest radiography is the most common imaging modality for pulmonary diseases. Due to its wide usage, there is a rich literature addressing automated detection of cardiopulmonary diseases in digital chest X-rays (CXRs). One of the essential steps for automated analysis of CXRs is localizing the relevant region of interest, i.e., isolating lung region from other less relevant parts, for applying decision-making algorithms there. This article provides an overview of the recent literature on lung boundary detection in CXR images.

**Methods:**

We review the leading lung segmentation algorithms proposed in period 2006–2017. First, we present a review of articles for posterior–anterior view CXRs. Then, we mention studies which operate on lateral views. We pay particular attention to works that focus their efforts on deformed lungs and pediatric cases. We also highlight the radiographic measures extracted from lung boundary and their use in automatically detecting cardiopulmonary abnormalities. Finally, we identify challenges in dataset curation and expert delineation process, and we listed publicly available CXR datasets.

**Results:**

(1) We classified algorithms into four categories: rule-based, pixel classification-based, model-based, hybrid, and deep learning-based algorithms. Based on the reviewed articles, hybrid methods and deep learning-based methods surpass the algorithms in other classes and have segmentation performance as good as inter-observer performance. However, they require long training process and pose high computational complexity. (2) We found that most of the algorithms in the literature are evaluated on posterior–anterior view adult CXRs with a healthy lung anatomy appearance without considering challenges in abnormal CXRs. (3) We also found that there are limited studies for pediatric CXRs. The lung appearance in pediatrics, especially in infant cases, deviates from adult lung appearance due to the pediatric development stages. Moreover, pediatric CXRs are noisier than adult CXRs due to interference by other objects, such as someone holding the child’s arms or the child’s body, and irregular body pose. Therefore, lung boundary detection algorithms developed on adult CXRs may not perform accurately in pediatric cases and need additional constraints suitable for pediatric CXR imaging characteristics. (4) We have also stated that one of the main challenges in medical image analysis is accessing the suitable datasets. We listed benchmark CXR datasets for developing and evaluating the lung boundary algorithms. However, the number of CXR images with reference boundaries is limited due to the cumbersome but necessary process of expert boundary delineation.

**Conclusions:**

A reliable computer-aided diagnosis system would need to support a greater variety of lung and background appearance. To our knowledge, algorithms in the literature are evaluated on posterior–anterior view adult CXRs with a healthy lung anatomy appearance, without considering ambiguous lung silhouettes due to pathological deformities, anatomical alterations due to misaligned body positioning, patient’s development stage and gross background noises such as holding hands, jewelry, patient’s head and legs in CXR. Considering all the challenges which are not very well addressed in the literature, developing lung boundary detection algorithms that are robust to such interference remains a challenging task. We believe that a broad review of lung region detection algorithms would be useful for researchers working in the field of automated detection/diagnosis algorithms for lung/heart pathologies in CXRs.

## Introduction

Chest radiography is one of the most common diagnostic imaging techniques for cardiothoracic and pulmonary disorders [1]. It is an early diagnosis tool that is commonly used in clinical settings to observe abnormalities in the cardiothoracic region which includes lung and heart pathologies, e.g., atelectasis, consolidation, pneumothorax, pleural and pericardial effusion, cardiac hypertrophy and hyperinflation [2]. It also serves as a valuable tool for tuberculosis (TB) screening for HIV+ population in resource-constrained regions [3–6]. Chest radiography is widely available, affordable, and has lower radiation dose compared to other imaging tools [1]. Particularly, under-resourced regions of the world that also have to face a heavy burden of infectious diseases, such as TB, commonly use chest X-ray (CXR) as frontline diagnostic imaging due to lower infrastructure setup, operational costs, and portability [7, 8]. Automated analysis of CXR can assist in population screening as well as the radiologist in triaging and interpretation, thereby reducing their workload [6, 9]. Further, they provide a valuable visual aid for the frontline clinician in diagnosing the patient. Also, automated analysis can help control inter-reader variability across radiologists, better discriminate abnormal cases for further expert interpretation, and even serve as a B-reader in the diagnostic decision-making process [10].

The typical steps in a conventional CXR analysis system include: (1) localizing the region of interest (ROI) (e.g., lung lobes) to focus the useful area for further processing; (2) extracting imaging features from ROI; and (3) applying a machine learning technique to detect/diagnose the abnormality [4, 11, 12]. Accurate localization of ROI impacts the performance of subsequent steps and the overall system. Therefore, it is an essential pre-processing stage in an abnormality detection/diagnostic process. With the recent resurgence of interest in artificial intelligence (AI), computer-aided detection/diagnosis systems have started to be developed with deep neural networks (DNNs) [13–15]. DNNs search abnormal patterns from the raw image data without setting explicit rules, detecting ROI, extracting features or user-in-the-loop intervention. However, DNNs are computationally expensive due to optimization of large number of model parameters which increase with image size. Therefore, restricting the processing area by removing background noise and processing only the relevant region becomes essential for improving the algorithm’s accuracy and lowering computational time in DNN-based approaches. In [16], researchers analyzed the impact of lung segmentation and bone shadow exclusion techniques in a DNN-based lung nodule detection algorithm. Higher training and validation accuracy are observed for segmented and bone shadow removed CXRs. Another recent DNN-based study applies histogram equalization and ROI detection before processing CXR images to increase the algorithm’s accuracy [17].

**Fig. 1:**
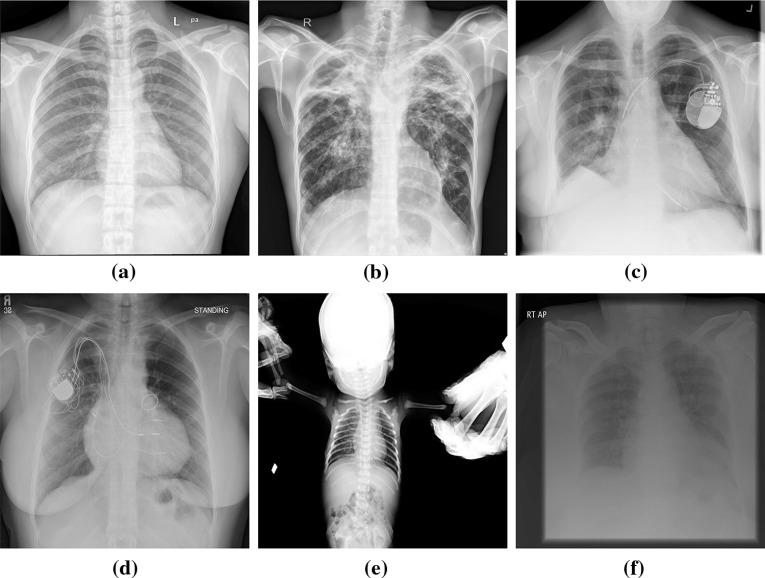
**a** A healthy lung. **b**–**f** Challenges for segmenting lung regions: **b** large variance of pixel values at apex due to pathology (bilateral tuberculosis with multiple cavitations), **c** a cardiac pacemaker, right pleural thickening, and strong breast tissue on the clavicle region of left lung, **d** variation of the lung appearance due to varying heart dimension, cardiac pacemaker on the left, and strong breast tissue on the clavicle regions, **e** image noise in pediatric CXR such as hands and patient’s head; small lung area, **f** an under-penetrated radiograph

For pulmonary diseases, the objective ROI is the lung region within the thorax. However, lung region detection for posterior–anterior (PA) CXRs is a well-studied problem (c.f. “Lung boundary detection in posterior–anterior CXR” section). Most of these algorithms are evaluated on adult CXR images with *“normal”* or unaltered lung anatomy appearance. The pathology and anatomical alterations can impact the intensity distribution in lung regions and result in ambiguous lung silhouettes which introduce challenges for automated border delineation algorithms. In addition to the lack of lung region detection algorithms robust to pathological deformities, the studies on pediatric CXRs (c.f. “Pediatric chest radiography” section) are limited in the literature. The lung appearance in pediatrics, especially in infant cases, deviates from adult lung appearance due to the pediatric development stages [18–20]. Therefore, a lung boundary detection algorithm developed on adult lungs may not accurately perform in pediatric cases [20]. Moreover, pediatric CXRs are noisier than adult CXRs due to holding hands, patient’s head, legs positioning, and rotation (Fig. 1e), which increases the importance of localizing the ROI and processing within it.

Considering all these challenges that are not very well addressed in the literature, developing lung boundary detection algorithms that are robust to pathological deformities, drastic shape irregularities, CXR orientation, CXR projection (posterior–anterior (PA), anterior–posterior (AP), lateral), and gross background noise in thoracic cavity remains a challenging task. We believe that a broad review of lung region detection algorithms would be useful for researchers working in the field of automated detection/diagnosis algorithms for lung/heart pathologies in CXRs. The paper is organized as follows. First, methods developed for PA-view CXRs are described in “Lung boundary detection in posterior–anterior CXR” section, and studies which include lateral-view CXRs are discussed in “Lung boundary detection in lateral view” section. We mention lung boundary detection algorithms for deformed lungs in “Lungs with deformed appearance” section and pediatric studies in “Pediatric chest radiography” section. The deviation in lung silhouette could be used as visual signs of abnormality and can be an additional feature for pathology detection/diagnose. In “Radiographic measures: radiological signs for pulmonary abnormalities” section, we survey studies which extract radiographic measurements from lung boundaries and make a diagnostic decision from these measurements. Finally, we list the main evaluation metrics for measuring lung region detection algorithms performance in “Evaluation of lung region detection algorithms” section and publicly available CXR datasets in “DataSets”section.

## Lung boundary detection in posterior–anterior CXR

Lung boundary detection in a CXR image can be thought of as two types of processes: (1) rule-based edge detection, where the edge belongs to the lung boundary; or (2) cast as a binary classification (region detection), where the goal is to label each pixel in the image as belonging to the lung region or background. There are several challenges in segmenting lung region in a CXR, which are depicted in Fig. 1, such as (1) lung appearance variations due to age, gender, heart dimension, pathology, and genetic variations between patients; (2) pixel intensity difference within the lung at hilum, apex, clavicle, and rib regions; (3) imaging inhomogeneities due to various breath states; (4) patient position during scanning; and (5) foreign objects such as implanted devices, buttons on patient clothes. Lung boundary detection in PA CXR is a well-studied problem. Earlier works have been reviewed in [21]; more recent methods are compared in [11] on a public dataset. However, these articles contain studies before 2001 and before 2006. In this study, we update an understanding of the field and review the studies published in period 2006–2018. Shi et al. [22] classified the segmentation algorithms into the following groups: (1) rule-based methods, (2) pixel classification methods, (3) deformable-based methods, and (4) hybrid methods. We adopt the same classes in this study. Although deep learning techniques can be listed in pixel classification methods, we consider them as a separate group due to their surpassing performance in computer vision.

### Rule-based methods

The algorithms in this group set sequential steps and heuristic assumptions to locate the lung region. They are generally used as initialization of more robust segmentation algorithms. For example, in [23], researchers propose using level sets which combine the global statistics, prior shape, and edge information. The level set is initialized at a seed mask which is computed using rule-based steps such as thresholding, morphology, and connected component analysis. In [24], lung region is extracted using Euler number method and refined through morphological operations. In [25], before applying fuzzy C-means clustering algorithms, sequential steps are applied such as Gaussian derivative filtering, thresholding, border cleaning, noise removal, and clavicle elimination. Several earlier approaches in this group are mentioned in [11] and in [26]. The algorithms in this group have an easier implementation. However, the output boundaries obtained with this algorithms may not be optimal due to sequential steps, e.g., applying morphological operations, resulting in cascaded accumulation of errors.

### Pixel classification-based methods

In these algorithms, each pixel is labeled as a lung or a non-lung pixel using a classifier (e.g., support vector machines, neural networks) that is trained with example CXRs and their corresponding lung masks. For example in [11], the proposed method employs multiscale filter bank of Gaussian derivatives and *k*-nearest neighbor (*k*-NN) classifier. The limitation of the conventional classification approaches is the lack of model constraint to keep the boundary in the expected lung shape. The classifier might fail at segmenting lung with lesions or other pathology without a reference model due to the difference in imaging characteristics in these areas.

### Model-based methods

The algorithms in this group use both low level appearance and shape priors. The earliest model-based algorithms are Active Shape Model (ASM) [27] and Active Appearance Model (AAM) [28] in which the shape is modeled with the distribution of landmark points on training images and is fitted to the test image by adjusting the distribution parameters. They are applied to lung region detection in [11, 29]. Despite their broad applicability due to shape flexibility, ASM and AAM do not perform well at widely varying shapes, require proper initialization for a successful convergence, and output boundary strongly rely on tuning the parameters. For lung region segmentation, the algorithm can get trapped at local minima due to strong rib cage and clavicle bone edges.

Several studies have been proposed as an extension of ASM and AAM to cope with their disadvantages by incorporating prior shape statistics in objective functions [30–33]. For example, in [22] the lung boundary is characterized by a scale-invariant feature transform, and ASM is constrained by statistics collected from previous CXRs of same and other patient’s CXRs. In [34], a shape particle filtering approach is used to prevent getting trapped at a local minimum. In [35], global edge and region forces are added as additional terms to the objective function to reach the global minimum.

### Hybrid methods

In these methods, the best parts of the schemes are combined to produce a better approach to overcome the challenges of lung boundary detection. For instance, in [11], deformable models and pixel classification approach are combined with majority voting, and a better boundary detection performance is reported. In [36], an atlas-based approach is used in which the model atlases are registered to the patient CXR using the SIFT-flow algorithm [37] and combined with graph cut boundary detection.

### Deep learning methods

With advances in GPU technology, computer vision systems designed with deep neural networks trained on a massive amount of data have been shown to produce more accurate results than conventional approaches. In deep neural networks, input data is processed through deep convolutional layers, which learn feature representation hierarchically, starting from low-level to more abstract representations. In particular, convolutional neural networks (CNNs) have received considerable attention in image analysis problems, since they preserve the spatial relationship between the image pixels.

Despite the popularity of deep learning algorithms in medical imaging, only a few studies have been reported in the literature for lung boundary detection in CXRs. A recent study uses semantic segmentation approach [38] in which the input is a CXR image and output is a map indicating lung region probability of each pixel. In [39], researchers proposed using fully convolutional networks (FCN) [40] for segmenting lung, clavicle and heart regions. FCN is an encoder-decoder architecture. The encoder models the semantic information in the image; the decoder recovers the location information which is lost during the pooling process and produces a map contains lung region probability of each pixel. FCN produces rough map due to its basic decoder architecture. Therefore, researchers [39] applied architectural modifications by adding a drop out layer after every convolutional layer, by re-ordering the feature maps and by replacing pooling layers with convolutional layers. In [41] SegNet [42], performance is investigated for lung region detection in CXRs. SegNet is a semantic segmentation approach which has similar encoder-decoder architecture as in FCN. However, each deconvolutional layer in the decoder stage corresponds to a convolutional layer at the same level; upsampling is performed based on the pooling indices in the corresponding encoder stage which provides more accurate segmentation map compared to FCN. In [43], researchers proposed using generative adversarial network (GAN) [44] for lung boundary detection in CXRs. GANs consist of two networks: a generator and a discriminator. For segmentation problem, the generator produces artificial lung masks using manually delineated lung regions; the discriminator produces probability if the mask is synthetic or it is from ground-truth mask set. Based on the probability, the discriminator guides the generator to generate masks more similar to the ground-truth masks.

All proposed DNN-based approaches perform as good as inter-observer performance for lung regions detection. The advantages and disadvantages of algorithms (as in groups) are summarized in Table 1. Quantitative comparisons of lung boundary detection algorithms are given in Table 2.

**Table 1:** Summary of advantages and disadvantages of the approaches for lung boundary detection algorithms in CXR images

Algorithm	Advantages	Disadvantages
Rule-Based Methods	Easy to implement	Produce rough solutions
[23–25]	Sets sequential steps	Generally used as initialization of robust approaches
	Lower computational complexity	Poor generalization capability
Pixel classification		Based on low-level features
[11]		Lack of shape constraints
Deformable Models	Provides shape flexibility	Do not perform well at widely varying shapes
[30–33]	Combines both low-level features and general shape of the lung	Require proper initialization for a successful converge
[22, 34, 35]		The possibility of trapping at local minimum due to bone intensity
Hybrid Methods	Best part of the schemes are combined	Might require long training process
[11, 36, 45]	Similar accuracy as in inter-observer accuracy	
Deep Learning Methods	Similar accuracy as in inter-observer performance	Long training process
[39, 41, 43]		Needs large set of annotated data
		Higher computational complexity

**Table 2:** Quantitative comparison of lung boundary detection algorithms

Authors, citation	Methology	Dataset	$\varOmega$	DSC	ACD
Ginneken et al. [11]	Human observer	JSRT	$0.946 \pm 0.018$	NA	$1.64 \pm 0.69$
Saad et al. [24]	Rule-based	CXR	0.809	NA	NA
Annangi et al. [23]	Deformable	CXR	$0.880 \pm 0.07$	NA	NA
Shi et al. [22]	Deformable	JSRT	$0.920 \pm 0.031$	NA	$1.78 \pm 0.78$
Coppini et al. [45]	Classification	JRST	$0.927 \pm 0.033$	$0.95 \pm 0.037$	$1.730 \pm 0.870$
Seghers et al. [46]$^1$	Deformable	JRST	$0.939 \pm 0.031$	NA	$1.49 \pm 0.63$
Candemir et al. [36]	Hybrid	JSRT	$0.954 \pm 0.015$	$0.967 \pm 0.008$	$1.321 \pm 0.316$
Candemir et al. [36]	Hybrid	NLM	$0.941 \pm 0.034$	$0.960 \pm 0.018$	$1.599 \pm 0.742$
Dawoud [30]	Deformable	JRST	$0.940 \pm 0.053$	NA	$2.460 \pm 2.060$
Novikov et al. [39]	Deep learning	JRST	0.950	0.974	NA
Shao et al. [26]	Hybrid	JRST	$0.946 \pm 0.019$	$0.972 \pm 0.010$	$1.699 \pm 0.762$
Kaur et al. [47]	Deep Learning	JSRT	0.934	NA	NA
Kalinovsky et al. [41]	Deep Learning	JSRT	NA	$0.962 \pm 0.008$	NA
Li et al. [48]	Deformable	JSRT	$0.931 \pm 0.018$	$0.964 \pm 0.010$	NA
Lee et al. [49]	Deformable	JSRT$^2$	$0.854 \pm 0.049$	NA	NA
Wu et al. [50]	Deformable	JSRT$^2$	$0.952 \pm 0.019$	NA	NA
Ibragimov et al. [51]	Classification	JSRT	$0.953 \pm 0.02$	NA	$1.43 \pm 0.85$
Yang et al. [52]	Classification	JSRT	$0.952 \pm 0.018$	$0.975 \pm 0.01$	$1.37 \pm 0.67$
Hwang et al. [53]	Deep learning	JSRT	$0.961 \pm 0.015$	$0.980 \pm 0.008$	$1.237 \pm 0.702$

$^{1}$Right lung scores, $^{2}$Subset of JSRT

$\varOmega$ Jaccard similarity coefficient, *DSC* dice similarity coefficient, *ACD* average contour distance, (See “Evaluation of lung region detection algorithms” section for metric descriptions), *CXR* non-public dataset. *NA* the respective metric is not reported in the publication

## Lung boundary detection in lateral view

**Fig. 2:**
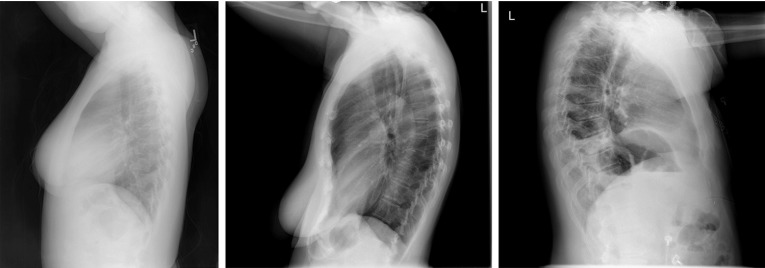
Example lateral-view CXRs

$15\%$ of the lung is not clearly visible in PA view because of the cardiovascular structure and the diaphragm [1]. Therefore, radiologists include lateral chest radiograph, when relevant, in their decision-making process [54]. Although they are routinely used in clinical decision-making, few automated schemes are reported in the literature that include lung region detection in lateral-view CXRs. One of the earlier algorithms which uses both frontal and lateral views is in [55, 56] for automatically assessing the costophrenic angle blunting. CXRs are segmented with iterative global and local thresholding followed by polynomial curve fitting for boundary smoothing. In [57], researchers developed an automated computer-based method for the calculation of total lung capacity by determining the pulmonary contours from PA and lateral CXRs. The lung borders are computed using lung shape profiles and thresholding. The edges are then completed using curve fitting techniques. A recent effort [45] was aimed at the automated computation of emphysema utilizing the shape of lung fields in both frontal and lateral chest radiographs. The lung boundary is modeled as a closed fuzzy curve and estimated by self-organizing networks [58] (Fig. 2).

## Lungs with deformed appearance

**Fig. 3:**
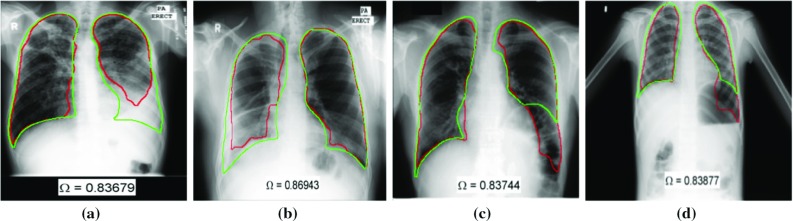
Example deformed lungs from NLM-Montgomery dataset [59]. Green contour is expected lung anatomy delineated by a radiologist [36]. Red contour is the algorithm’s decision as lung boundary. **a**, **b** The algorithm could not detect the lung boundary correctly due to opacity caused by fluid in the lung space. **c** The left diaphragm is elevated, and there is a large air-distended colon loop below the lung which is incorrectly combined with the lobe into a single region by the algorithm. **d** Detected lung boundary includes the air cavity below left lung

The lung boundary detection in PA chest radiograph is a well-explored problem. However, most of the algorithms in the literature are evaluated on CXRs with *“normal”* lung anatomy appearance, i.e., without structural deformities. A reliable computer-aided diagnosis (CAD) system would need to support a greater variety of lung shapes, deformed/occluded due to disease, accidents, or postsurgical alterations, e.g., pneumonectomy or lobectomy. Pathology and anatomical alterations impact the intensity distribution in the lung region, deform the lung anatomy shape, or result in an ambiguous lung silhouette. In addition to textural and shape deformations in lung appearance, the regions outside the lung might appear like part of the lung (e.g., stomach gas). As in Fig. 3, the algorithm’s decision [36] for lung boundary (red contour) is significantly different from the expected lung anatomy (green contour delineated by an expert). The missing parts may contain important clues about the abnormality and could be useful for algorithm’s decision [5]. Therefore, automated lung boundary detection algorithms that are robust to cardiopulmonary deformities in thoracic cavity remains a challenging task.

Most of the algorithms in the literature were developed and evaluated on the Japanese Society of Radiological Technology (JSRT) dataset [60] (c.f. “Publicly available CXR datasets” section) since the dataset and its reference boundaries [11] were the only well-known publicly available set until 2015. However, JSRT dataset is curated for developing lung nodule detection algorithms; the radiographs do not contain abnormalities which cause lung shape and texture deformation. Recently, a new CXR dataset [59] and their lung boundaries [36] were made publicly available by the U.S. National Library of Medicine (NLM). This set contains deformed lung appearance (both shape and tissue) due to the manifestations of tuberculosis (c.f. “DataSets” section). There are only a few studies that have evaluated the lung boundary detection algorithms on deformed lungs. In [36], a model-based algorithm is tested on NLM’s Montgomery dataset. However, the performance of this approach relies on the patient CXR being well-modeled by the training lung masks. Therefore, the algorithm might fail at large deformed lung shapes, if a similar mask is not present in the training set. In [33], researchers proposed an ASM-based method in which the shape prior is incorporated with a selective thresholding algorithm. The algorithm is initialized at salient points (spinal cord and ribcage) which are robust to pulmonary abnormalities. The method’s accuracy is evaluated on portable chest radiographs with deformed lung appearance.

### External objects

In addition to intra-thoracic pathology, lungs appearance is often distorted by external objects that may be present due to poor quality assurance, e.g., jewelry, buttons [61, 62], body piercings, or external elements due to patient age, e.g., cardiac pacemaker, tubes [61]. Examples of some of these distortions are shown in Fig. 1c–e. Such a distorted appearance can distract the algorithm and lead to inaccurate segmentation. Although there are articles recognize the importance of such distortions [61, 63, 64], to our knowledge, there is not any methodical inclusion of these challenges into a lung segmentation algorithm that is robust to such real-world image artifacts.

### Subject positioning

A significant problem in developing robust lung segmentation algorithms is patient positioning. Most algorithms found in the literature assume that the patient is upright with appropriately inflated lungs and properly positioned without rotation. However, real-world CXR images, particularly those from hospital settings or of physically disabled subjects, have these problems. Subject positioning lead to deformed lung appearance, thus adversely impact the lung segmentation stage and subsequent decision-support algorithms. Some articles in the literature [25, 63, 64] attempt to correct planar rotation, which is important for image analysis, but we do not find articles that detect patient rotation to aid in improved imaging quality assurance.

## Pediatric chest radiography

According to the 2015 RAD-AID Conference on International Radiology for Developing Countries report [7], approximately 3 to 4 billion people in the world do not have easy access to radiology services; among them, approximately 500 million to 1 billion are children. Therefore, RAD-AID and International Day of Radiology [65], an annual event supported by the European Society of Radiology, the American College of Radiology, and the Radiological Society of North America, have started to emphasize the importance of pediatric radiology [7]. Chest radiography is a valuable diagnostic tool in the pediatric population, and it is a key diagnostic tool in TB detection for pediatric patients in low-burden countries, due to the lower sensitivity of TB culture test (current gold standard for active TB detection) in pediatrics [66]. To our knowledge, only a few computerized methods have been developed for pediatric CXRs. In [19, 67], researchers propose a CAD system for pulmonary pathology in pediatric CXRs and use ASM [27] to segment the lung regions. ASM requires proper initialization for a successful convergence (c.f. “Model-based methods” section). Therefore, researchers initialize the algorithm by manually marking the distinct anatomical locations in each lung field. In [20], researchers characterized the shape differences between age groups and enhanced their fully automated model-based approach [36] toward pediatric lung boundary detection by building age-based lung training models. One of the recent efforts utilized a deep learning approach to estimate the statistical shape model parameters and applied the algorithm for lung region detection in pediatric CXRs [68].

**Fig. 4:**
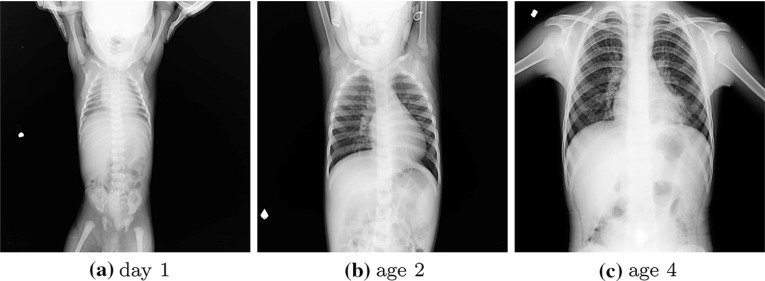
Example pediatric CXRs and visible differences between lung appearance due to pediatric development stages

Pediatric chest radiography has distinct challenges compared to adult chest radiography. The lung appearance between age groups has visible differences due to pediatric development stages [18, 19] (Fig. 4). In an infant, lungs are smaller, have a triangular shape, and the cardiac silhouette is relatively larger such that the horizontal diameter of the heart may approach $60\%$ of thoracic horizontal diameter [18]. Besides, pediatric CXRs have distinct background noise such that high frequency of mother’s holding hands, patient’s head, and legs (Figs. 1e, 4a). Due to the visible appearance difference between adult and pediatric CXRs along with additional challenges in pediatric chest radiography, lung boundary detection algorithms developed on adult lungs may not perform well on pediatric cases [20].

## Radiographic measures: radiological signs for pulmonary abnormalities

  Some lung pathologies such as consolidation, atelectasis, and pleural effusion are clearly visible on CXRs due to appearance deformation within the lung region. The deviation in lung silhouette could be used as visual signs of abnormality and can be an additional feature for pathology detection/diagnosis. In this section, we survey studies which make a diagnostic decision from the radiographic measures extracted from lung boundaries.

**Fig. 5:**
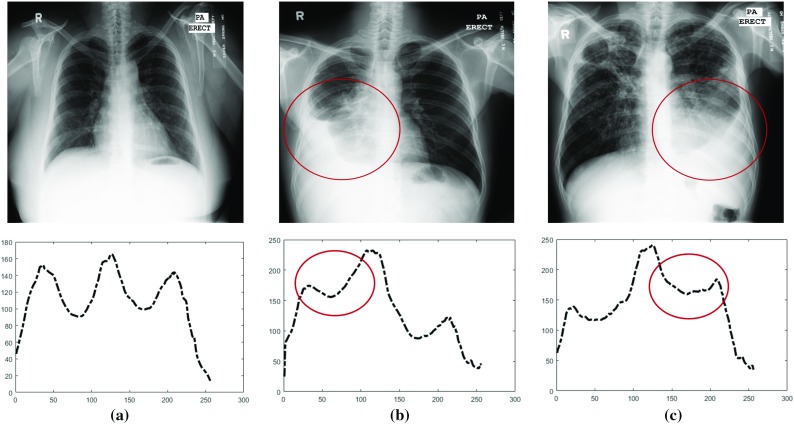
Illustration of lung shape profiles computed by summing up pixels in each column. **a** A healthy lung. **b** Pleural effusion on the right lung due to tuberculosis. **c** Pleural effusion on the left lung. *Note*: Circled area in chest X-rays and histogram alteration in pathological regions

One of the structural information extracted from lung boundary is *CXR shape profiles* which is the intensity value distribution in horizontal and vertical directions, obtained by summing up pixel intensities in each column and row. Fig. 5 illustrates the horizontal lung shape profiles of example CXRs. Despite their simplicity, lung shape profiles provide strong shape features. For example, pleural effusion, which is associated with congestive heart failure and TB, is a whitening area on lung caused by radiological opacity due to accumulated fluid in the pleural cavity [2]. Figure 5b, c shows example CXRs with pleural effusion and their corresponding lung shape profiles. Note the histogram’s dissimilarity between healthy and non-healthy lungs. Besides, lung shape profiles are used as a rough lung region detection scheme as in [57, 69] with peak analysis of profile histograms and additional feature for frontal/lateral CXR classification [70].

Several other shape features that can be extracted from lung boundaries such as size, orientation, eccentricity, extent, and centroid coordinates. In [5], researchers extract low-level shape features and combine them with texture features to increase the TB detection performance. The area under the curve (ROC) in detecting TB increased by 2.4% with shape features addition. In [71], the method computed *lung region symmetry* features in addition to low-level shape features; and measured their contribution to the TB detection.

**Fig. 6:**
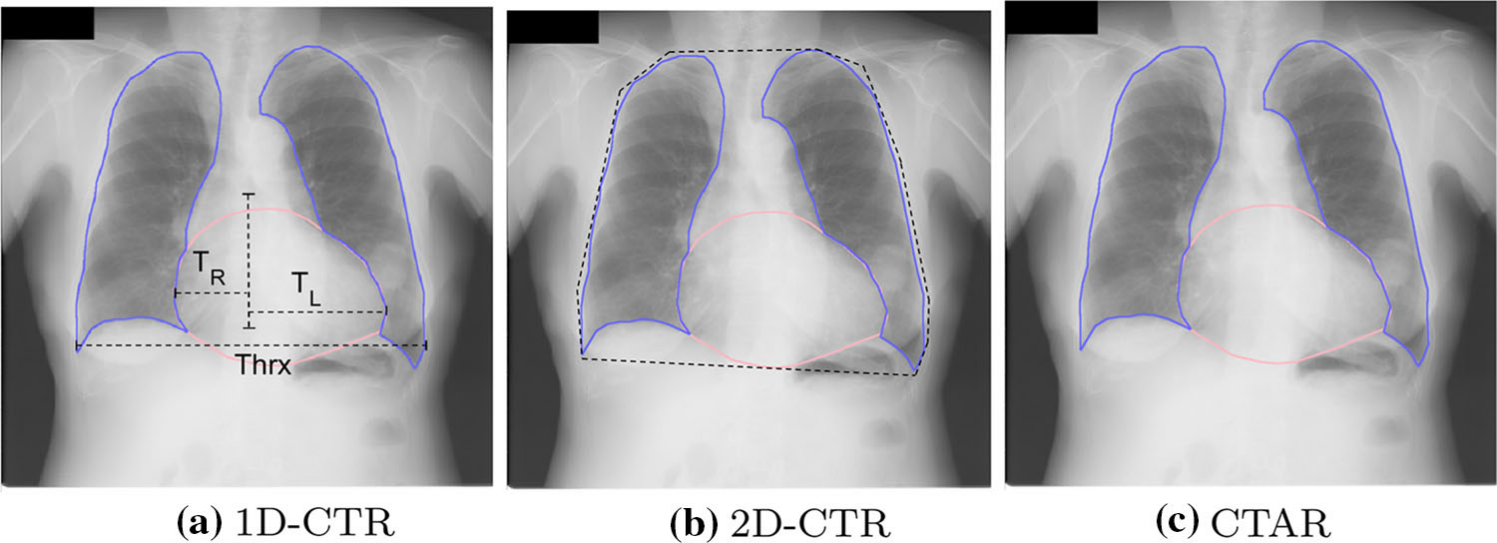
Illustration of radiographic index computation using lung and heart boundaries on CXR

One of the structural abnormalities that can be observed in CXRs is *emphysema*, which is the hyperinflation of the alveoli, affects the lung silhouette appearance [45]. In [45, 72, 73], researchers utilized geometrical features extracted from lung boundaries to automatically detect emphysema. The other structural abnormality is *cardiomegaly* which is a medical condition caused by high blood pressure or coronary artery disease. The literature has several studies which extract radiographic indexes from lung boundary and use them for early detection of heart diseases [11, 22, 31, 74]. The clinically used measurement is cardiothoracic ratio (CTR) which is defined as the ratio between the maximum transverse cardiac diameter and the maximum thoracic diameter measured between the inner margins of the ribs [75] (Fig. 6a). The other radiographic indexes suggested as an alternative to CTR are 2D-CTR [76] and CTAR [69]. 2D-CTR is the ratio between the pixel counts of the cardiac outline and whole thorax (Fig. 6b), and CTAR [69] is the ratio of the area of heart region to the area of lung region (Fig. 6c). Accurate lung and heart boundary information are critically important in computing radiographic indexes. In studies [11, 22, 31], CTR computation is proposed as a clinical application of anatomical boundary detection methods. The cardiomegaly detection performance of radiographic indexes in the literature are compared in [74] on a publicly available dataset. In [77], performance of radiographic indexes are compared with data-driven approaches on the same public dataset.

## Evaluation of lung region detection algorithms

There are several metrics to evaluate the performance of lung boundary detection algorithms. Roughly, metrics are divided into two classes: (1) overlap-based metrics and (2) distance-based metrics [78].

Overlap metrics quantify the overlapping area between the algorithm’s segmentation and reference boundaries. The most widely used one is Jaccard similarity coefficient,

1 $$\begin{aligned} \varOmega = \frac{|\mathrm{TP}|}{|\mathrm{FP}|+|\mathrm{TP}|+|\mathrm{FN}|} \end{aligned}$$

where TP (true positives) represents correctly classified pixels, FP (false positives) represents background pixels that are classified as lung, and FN (false negatives) represents lung pixels that are classified as background. The other overlapping measure is Dice similarity coefficient [79] formulated as follows,

2 $$\begin{aligned} \mathrm{DSC} = \frac{2 |\mathrm{TP}|}{2|\mathrm{TP}| + |\mathrm{FN}| + |\mathrm{FP}|}. \end{aligned}$$

Both measures have a value between 0 and 1; 1 indicates fully overlapped segmentation.

Overlapping metrics are based on correctly or incorrectly classified pixels. The classification value of each pixel has the same impact to the computation regardless of their distance to the reference border [78]. Therefore, overlapping metrics alone are not sufficient to evaluate the region detection algorithm’s performance. Researchers use distance-based measures such as *average contour distance (ACD)* which quantifies how far apart the reference lung boundary and algorithm’s estimated boundary are from each other. Let $a_i$ and $b_j$ are the points on the algorithm’s detected boundary *S* and reference boundary *R*, respectively. The minimum distance of point $a_i$ on *S* to the reference boundary *R* is defined as follows,

3 $$\begin{aligned} d(a_i,R) = \mathrm{min}_j|| b_j-a_i||. \end{aligned}$$

ACD measures the minimum distance of each point on the boundary *S* to the contour *R*. The distances are averaged over all points of boundary *S*. To make the similarity measure symmetric, the computation is repeated from reference contour to the algorithm’s estimated contour. ACD is formulated as follows,

4 $$\begin{aligned} \mathrm{ACD} = \frac{1}{2} \left( \frac{\sum _{i} d(a_i,R) }{ |\{ a_i \}|} + \frac{\sum _{j} d(b_j,S)}{ |\{ b_j \}|}\right) \end{aligned}$$

where $| \cdot |$ is the cardinality of the set.

## DataSets

### Curating datasets

One of the main challenges in medical image analysis is access to suitable datasets. It is usually difficult to avail of appropriately sized de-identified data that can be used for algorithm development. Further, curated datasets are generally clean and may not reflect normal variations in image acquisition characteristics (e.g., device, subject positioning, exposure, resolution), appropriate distribution of diseases that reflect their prevalence, adequate distribution among various age groups, or reflect the gender diversity. Further, the images are modified such that they are windowed or leveled for human visual analysis. They are rarely accompanied with full clinical reports or at least pertinent sections of the reports such as the radiologists’ impressions and readings. Finally, image datasets are often not in the original DICOM format as acquired at the clinical sites. It is desirable that datasets be available that address the above and include expert delineation of important organs and zonal markup data indicating the location of disease. All of these characteristics are partially addressed in the datasets identified below, but each lacks some key element that could hamper advances in the field.

### Expert delineation of reference boundaries

In order to train and evaluate the system performance of automated lung boundary detection algorithms, reference lung boundaries are needed. However, expert delineation which is a task that is unnatural for domain experts, i.e., radiologists, is cumbersome, slow, and prone-to-error. User-friendly interactive annotation toolboxes such as Firefly [80, 81] or LabelMe [82] may ease the delineation and speed up the process. For instance, in [36], reference lung boundaries are manually delineated by an expert by clicking points along the lung border (by considering the lung anatomy) through Firefly which is web-based interactive labeling tool [80, 81].

Although reference boundaries are used for training and evaluation, expert delineation introduces high inter- and intra-observer variabilities because of the subjective nature of the delineation process [78]. For instance, in study [5], two radiologists delineate the lung boundaries on the same CXRs. The inter-observer agreement is measured from the delineations. For normal lungs, inter-observer agreement is $(\mu , \sigma ) = (86\%, 13.6)$. However, for deformed lungs, the inter-observer agreement is $(\mu , \sigma ) = (73\%, 18.1)$, slightly lower than lung agreement for normal cases, mainly because of the invisible border occurred due to pathology. With the standardization of annotation guidelines and with the help of the interactive tools, the subjectivity of the delineation process may decrease.

### Publicly available CXR datasets

To our knowledge, there are few publicly available CXR datasets along with expert annotated lung boundaries and other characteristics identified above. Following are the list of these datasets.

*JSRT dataset* [60] is compiled by the Japanese Society of Radiological Technology (JSRT) which contains 247 CXRs (154 CXRs with lung nodules and 93 CXRs without lung nodules). All CXRs have a size of $2048 \times 2048$ pixels, the spatial resolution of 0.175 mm/pixel and 12-bit grayscale color depth. The CXRs are publicly available at [83]. In addition, patient age, gender, diagnosis, and the location of the anomalies are provided as text files. The reference lung boundaries (along with heart and clavicle boundaries) are available at [11, 84]. This dataset is collected for developing lung nodule detection algorithms. Therefore the only abnormality in this set is lung nodules which do not cause any shape and texture deformations on the lungs.

*NLM Sets* [59]: The U.S. National Library of Medicine has made two CXR datasets available: the Montgomery and Shenzhen datasets. *The Montgomery set* contains 138 frontal CXRs from Montgomery County’s Tuberculosis screening program. Eighty of the X-rays are normal, and 58 of X-rays have manifestations of TB. The size of the X-rays is either $4020 \times 4892$ or $4892 \times 4020$ with 12 bit grayscale color depth. The reference lung regions of CXRs are manually delineated by an expert radiologist [36]. The *Shenzhen set* is collected in collaboration with Shenzhen No.3 People’s Hospital, Guangdong Medical College, Shenzhen, China. The set contains 662 CXRs. Three hundred twenty-six of X-rays belong to normal cases, and 336 cases have manifestations of TB. CXR sizes vary but approximately $3\,\mathrm{K} \times 3\,\mathrm{K}$. The datasets are publicly available at [85].

*Belarus Set* [86] is collected for a drug resistance study initiated by the National Institute of Allergy and Infectious Diseases, the United Institute of Informatics Problems of the National Academy of Sciences of Belarus, and the Republican Research and Practical Center for Pulmonology and Tuberculosis, Ministry of Health, Republic of Belarus. Much of the data collected for this study is publicly available [86]. The set contains both CXRs and CTs of 169 patients. Chest radiographs were taken using the Kodak Point-of-Care 260 system with $2248 \times 2248$ pixel resolution. Reference boundaries of the lung regions are available for each CXR.

The literature has several other publicly available CXR databases such as NIH-CXR dataset [87], NLM Indiana CXR collection [88], and New Delhi dataset [89]. However, there are no reference lung boundaries for the CXRs in these sets.

## Future challenges

With improved imaging using CT or MRI, the question is often raised if CXRs remain relevant today for diagnosis? CXR analysis has been known to be a less desirable diagnostic imaging technique whether it is by radiologists or by a machine [90] due to its poor diagnostic sensitivity and specificity. Yet, it remains the most common diagnostic imaging technique for cardiothoracic and pulmonary disorders [1]. That is mainly because of lower infrastructure setup, operational costs, and radiation dose compared to other imaging techniques [1, 7]. The use of CXRs continues unabated particularly in lower resource settings which often face challenges of highly infectious diseases. Low-resource settings face not only shortages in imaging capability but also radiological expertise. For example, the World Health Organization observes that in Malawi in sub-Saharan Africa, a country heavily burdened by HIV and TB, there is limited radiologists in public service [91]. In such settings, machine learning-based screening and diagnostic tools on CXRs have the potential of making a significant public health impact. Further, CXR remains a common modality for pediatric imaging with referrals for CTs only if warranted and if available. In the light of these observations, we can assume that future work will continue to include automated CXR analysis though with increasing interest in high quality 3D CT data.

## Conclusions

Detecting lung lobes is a critical processing stage in the automated analysis of CXRs for pulmonary disorders. Accurate localization of the lung region and processing only the region of interest positively impacts the overall performance of the diagnosis/detection systems, augment its accuracy and efficiency. In this study, we provided an overview of the recent literature on lung boundary detection. We believe that such a broad review of lung region detection algorithms would be useful for researchers working in the field of automated detection/diagnosis algorithms for lung/heart pathologies in CXRs. Following are our conclusions:

- We first summarized lung boundary detection algorithms developed for posterior–anterior view CXRs. Due to the rich literature, we classified algorithms as rule-based, pixel classification-based, model-based, hybrid, and deep learning-based algorithms. Advantages and disadvantages of each class are listed in Table 1. We conclude that hybrid methods and deep learning-based methods (1) surpass the algorithms in other categories, (2) have segmentation performance as good as inter-observer segmentation performance, however, and (3) have long training process and high computational complexity.
- Based on the reviewed articles, we can assert that most of the algorithms in the literature are evaluated on posterior–anterior view adult CXRs with “normal” lung anatomy appearance, without considering ambiguous lung silhouette, pathological deformities, anatomical alterations, patient’s development stage, and gross background noises such as holding hands, patient’s head, and legs in CXR. However, a reliable CAD system would need to support a greater variety of lung shapes, deformed due to disease or postsurgical alterations. We can suggest researchers should focus on developing algorithms that are robust to pathological deformities, shape irregularities, CXR orientation, CXR projection view, and gross background noise in the thoracic cavity.
- The other challenging area that researchers could focus on is pediatric CXRs. The lung appearance in pediatrics, especially in infant cases, deviates from adult lung appearance due to the pediatric development stage. Therefore, a lung boundary detection algorithms developed on adult lungs may not accurately perform on pediatric cases. Moreover, pediatric CXRs are noisier than adult CXRs due to holding hands, patient’s head and legs, and rotation, which increases the importance of localizing the ROI and operating within it. We can conclude that algorithms which are developed/tested on adult lungs should incorporate additional constraints in their algorithms suitable to pediatric CXRs characteristics.
- Finally, we identify challenges in dataset curation and expert delineation process, and we listed publicly available CXR datasets. We can state that one of the main challenges in medical image analysis is accessing suitable datasets. We have listed benchmark CXR datasets to develop and compare lung boundary algorithms. However, due to the necessity of expert delineation and its cumbersome process, the number of CXR images with reference (radiologist delineated) boundaries are limited.
